# Significant and conservative long-range transport of dissolved organic nutrients in the Changjiang diluted water

**DOI:** 10.1038/s41598-018-31105-1

**Published:** 2018-08-24

**Authors:** Hyeong Kyu Kwon, Guebuem Kim, Jeomshik Hwang, Weol Ae Lim, Jong Woo Park, Tae-Hoon Kim

**Affiliations:** 1 School of Earth and Environmental Sciences/Research Institute of Oceanography, Seoul National University, Seoul, 08826 Republic of Korea; 2 Ocean Climate and Ecology Research Division, National Institute of Fisheries Science, Busan, 46083 Republic of Korea; 3 Department of Earth and Marine Sciences, Jeju National University, Jeju, 63243 Republic of Korea

## Abstract

The Changjiang River is one of the main nutrient sources in the northwestern Pacific marginal seas. However, most of the previous studies have neglected the long-range transport (>200 km) of riverine nutrients since they are rapidly consumed. In this study, we examined the long-range transport (200–800 km) of nutrients in the surface layer during the summer of 2017. The plots of nutrients against salinity display that dissolved organic nitrogen (DON) was conservative over ~800 km, while more than 99% of the dissolved inorganic nitrogen (DIN) was removed within 200 km. As a result, in the study region, DON concentrations (avg. 7.0 ± 1.3 µM), which are minor in the river water, were much higher than DIN concentrations (avg. 0.28 ± 0.26 µM). Both nutrients, N and P, showed a similar pattern. Our results suggest that dissolved organic nutrients play a critical role on the long-range transport of riverine nutrients in surface waters and subsequent ecosystem changes.

## Introduction

In the global ocean, the riverine inputs of nutrients (37–66 Tg N yr^−1^ and 4–11 Tg P yr^−1^)^1–3^ may rival the atmospheric depositional fluxes (67 Tg N yr^−1^ and 0.54 Tg P yr^−1^)^4,5^. The largest fluxes of dissolved inorganic nitrogen (DIN) and phosphorus (DIP) from major rivers include the Amazon River (1.16 Tg N yr^−1^ and 0.13 Tg P yr^−1^), Mississippi River (0.82 Tg N yr^−1^ and 0.05 Tg P yr^−1^), Changjiang River (0.59 Tg N yr^−1^ and 0.02 Tg P yr^−1^), Rhine River (0.36 Tg N yr^−1^ and 0.02 Tg P yr^−1^), and the Ob River (0.30 Tg N yr^−1^ and 0.03 Tg P yr^−1^)^2,6–10^. Approximately 80% of the dissolved riverine nutrients are known to reach the open ocean^11^. On the other hand, the riverine fluxes of dissolved organic nitrogen (DON, 0.22–1.13 Tg N yr^−1^) and phosphorus (DOP, 0.01–0.09 Tg P yr^−1^) are known to account for a minor portion of the dissolved total nutrients in the global ocean^2^.

The Changjiang River is one of the main nutrient sources in the northwestern Pacific marginal seas. Over the past 50 years, the riverine fluxes of DIN and DIP have increased approximately 6-fold due to the large-scale use of chemical fertilizers in the Changjiang River basin^12^. According to the DIN budget for the East China Sea^12–14^, the DIN flux from the Changjiang River (~1.54 Tg N yr^−1^) is larger than the atmospheric depositional flux (~0.7 Tg N yr^−1^). The rapid increase in anthropogenic nutrient supply often resulted in the shift from N-limited to P-limited conditions for biological production in the Korea/Tsushima Strait from 2007 to 2014^15^. On the other hand, the exports of suspended sediments were significantly decreased by approximately 50% following the construction of the Three Gorges Dam in 2003^12^, although nutrients can be enhanced by about 40% in the estuarine mixing zone due to desorption from sediments^16^. However, the long-range transport of dissolved organic nutrients in this region is poorly understood.

**Figure 1:**
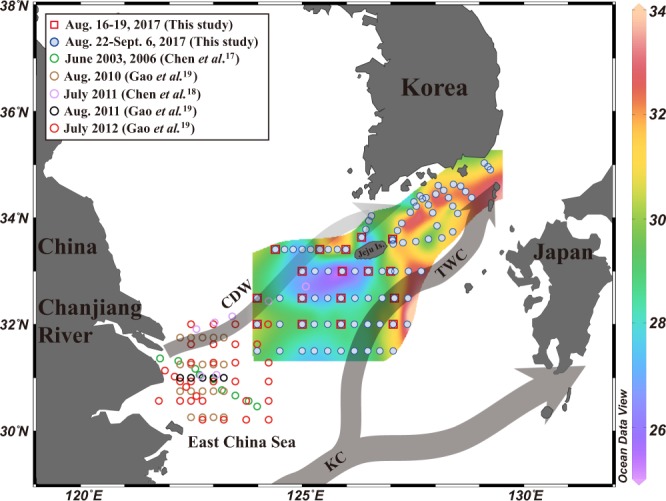
Map showing sampling stations and schematic patterns of surface currents in the northwestern Pacific marginal seas during the summer. The solid arrows represent the surface currents, such as the Changjiang diluted water (CDW), the Kuroshio Current (KC), and the Tsushima Warm Current (TWC) originating from KC. Open circles denote reference stations obtained from Chen *et al*.^17,18^ and Gao *et al*.^19^. Contour map denotes horizontal distribution of salinity in surface waters of the East China Sea from August 22 to September 6, 2017. The contour map was created using Ocean Data View software version 5.0.0. (Schlitzer, R., Ocean Data View, odv.awi.de, 2017) and the sampling stations and current patterns were drawn using Adobe Illustrator CS6 software version 16.0.0. (https://www.adobe.com).

Thus, in this study, we measured dissolved inorganic and organic nutrients in the downstream of the Changjiang estuary over a long distance (200–800 km) since most of the previous studies were conducted within 200 km^13,16–20^. Although it is ideal to include the entire salinity range from the river to 800 km in the study region, we could not arrange sampling cruises in both Chinese and Korean territories. Therefore, we occupied our study region (200–800 km) twice in the same season, and all previous data within 800 km were included in this study in order to look at the general processes occurring in this region (Fig. 1).

## Materials and Methods

### Study region

The study region is located in the northwestern Pacific Ocean, including the South China Sea, East China Sea, and East Sea (Sea of Japan) (Fig. 1). The primary source of freshwater in the East China Sea, which is one of the largest continental shelves in the world, is river runoff, and approximately 90% of the terrestrial materials entering are derived from the Changjiang River^18^. The discharge, which empties into the Changjiang estuary, forms a water type called the Changjiang diluted water (CDW), generally with salinity ≤31, by mixing with ambient seawater^21^. The Kuroshio branch water, which is a strong western boundary current and very oligotrophic, flows into the study region. Here, the branch of the Kuroshio Current mixes with the river waters, thereby obtaining large amounts of terrestrial organic matter and nutrients^17,18^. CDW flows southward along the Chinese coast in the winter, but in the summer it generally extends north-eastwards toward Jeju Island and even up to the East Sea^21,22^ (Fig. 1).

### Sampling and analyses

Surface seawater samples were collected during two periods in the East China Sea (Fig. 1) from August 16 to 19, 2017 on the R/V Ara of Jeju National University, Korea, and from August 22 to September 6, 2017 on the R/V Tamgu-3 and the R/V Tamgu-8 of National Institute of Fisheries Science (NIFS), Korea. Seawater samples were collected using Niskin bottles mounted on a CTD rosette sampler. Salinity was measured using a portable sensor (Orion star A329, Thermo Scientific).

Seawater samples for nutrients were filtered through pre-combusted (450 °C, 4 h) glass-fiber filters (Whatman GF/F, 47 mm in diameter, 0.7 µm in pore size) and stored frozen (−20 °C) until analysis. Dissolved inorganic nutrients, including NH_4_^+^, NO_2_^−^, NO_3_^−^, and PO_4_^3−^, were analyzed using a nutrient auto-analyzer (New QuAAtro39, SEAL Analytical). Reference seawater materials of nutrients (KANSO Technos, Tsukuba, Japan) were run for verification of analyses. The detection limits for NH_4_^+^, NO_2_^−^, NO_3_^−^, and PO_4_^3−^ were 0.03 µM, 0.01 µM, 0.02 µM, and 0.01 µM, respectively. In this study, the sum of NH_4_^+^, NO_2_^−^, and NO_3_^−^ is considered as DIN, and PO_4_^3−^ is considered as DIP. Total dissolved nitrogen (TDN) and phosphorus (TDP) analyses were determined using the method described by Grasshoff *et al*.^23^. Briefly, an acid potassium persulfate (N and P analysis grade, Wako) solution was added to a filtered seawater sample, and then autoclaved at 120 °C for 30 min. The resulting NO_3_^−^ and PO_4_^3−^ were measured with the auto-analyzer as described above. The accuracy of the TDN concentrations was verified using the deep-seawater reference material (32–33 µM; University of Miami, USA). The concentrations of DON and DOP were calculated by subtracting the measured DIN and DIP concentrations from the measured TDN and TDP concentrations, respectively.

## Results and Discussion

### Distributions of salinity and dissolved nutrients in the East China Sea

**Figure 2:**
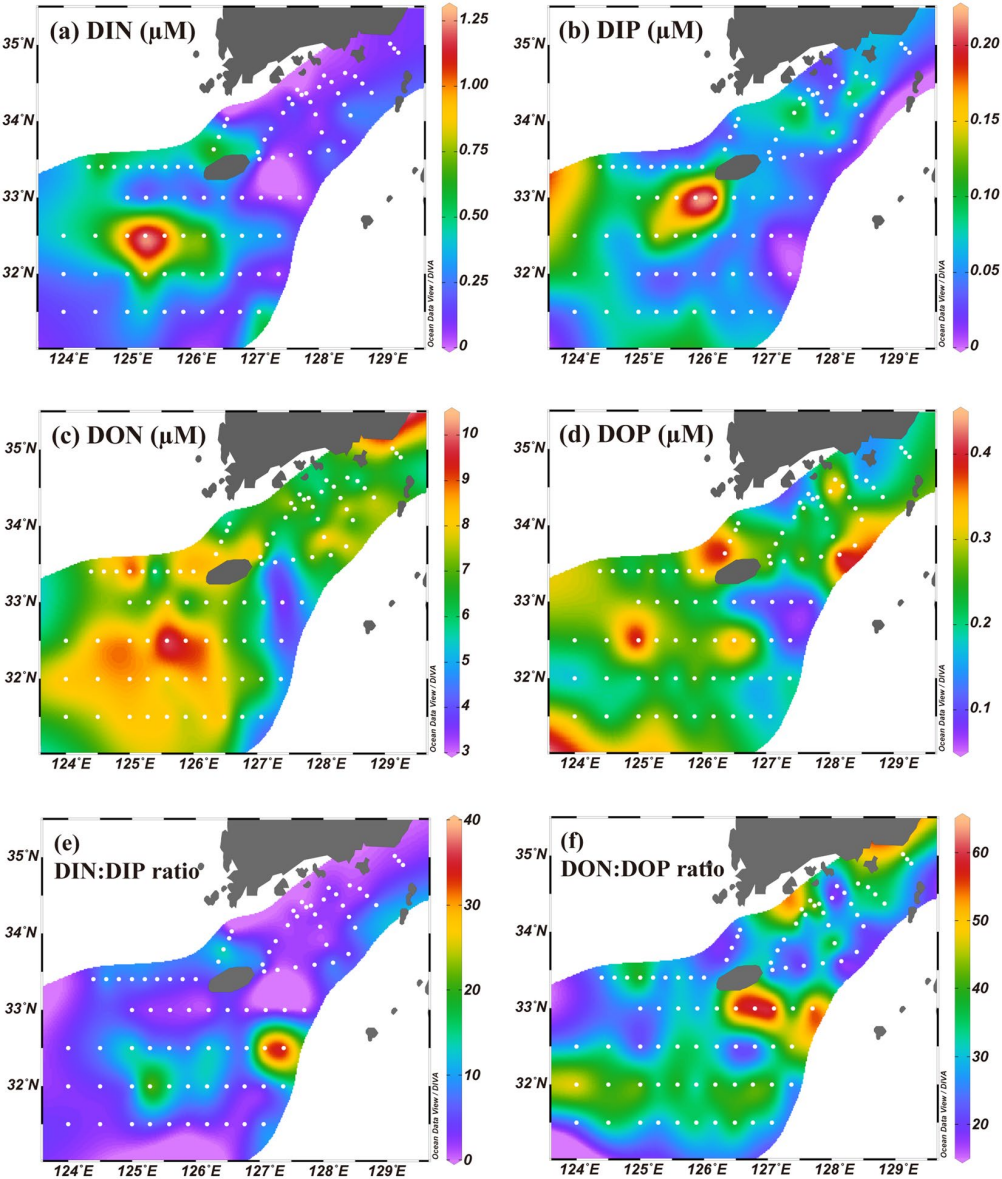
Horizontal distributions of (**a**) DIN, (**b**) DIP, (**c**) DON, (**d**) DOP, (**e**) DIN:DIP ratio, and (**f**) DON:DOP ratio in surface waters of the East China Sea from August 22 to September 6, 2017. The contour maps were created using Ocean Data View software version 5.0.0. (Schlitzer, R., Ocean Data View, odv.awi.de, 2017).

The salinity ranged from 25.33 to 33.99 (avg. 29.79 ± 1.99) in the surface waters of the study region (Supplementary Table S1). The salinity distribution in the surface waters shows that the low-salinity (<31) water is widely distributed over the East China Sea (Fig. 1), due to the influence of CDW^24^. A patch of low-salinity water (<28) was observed in the southwestern region of Jeju Island, which seems to be detached from the Changjiang plume^21,25^. In general, higher concentrations of nutrients were observed in the lower salinity areas, including the low-salinity patch area (Fig. 2).

**Figure 3:**
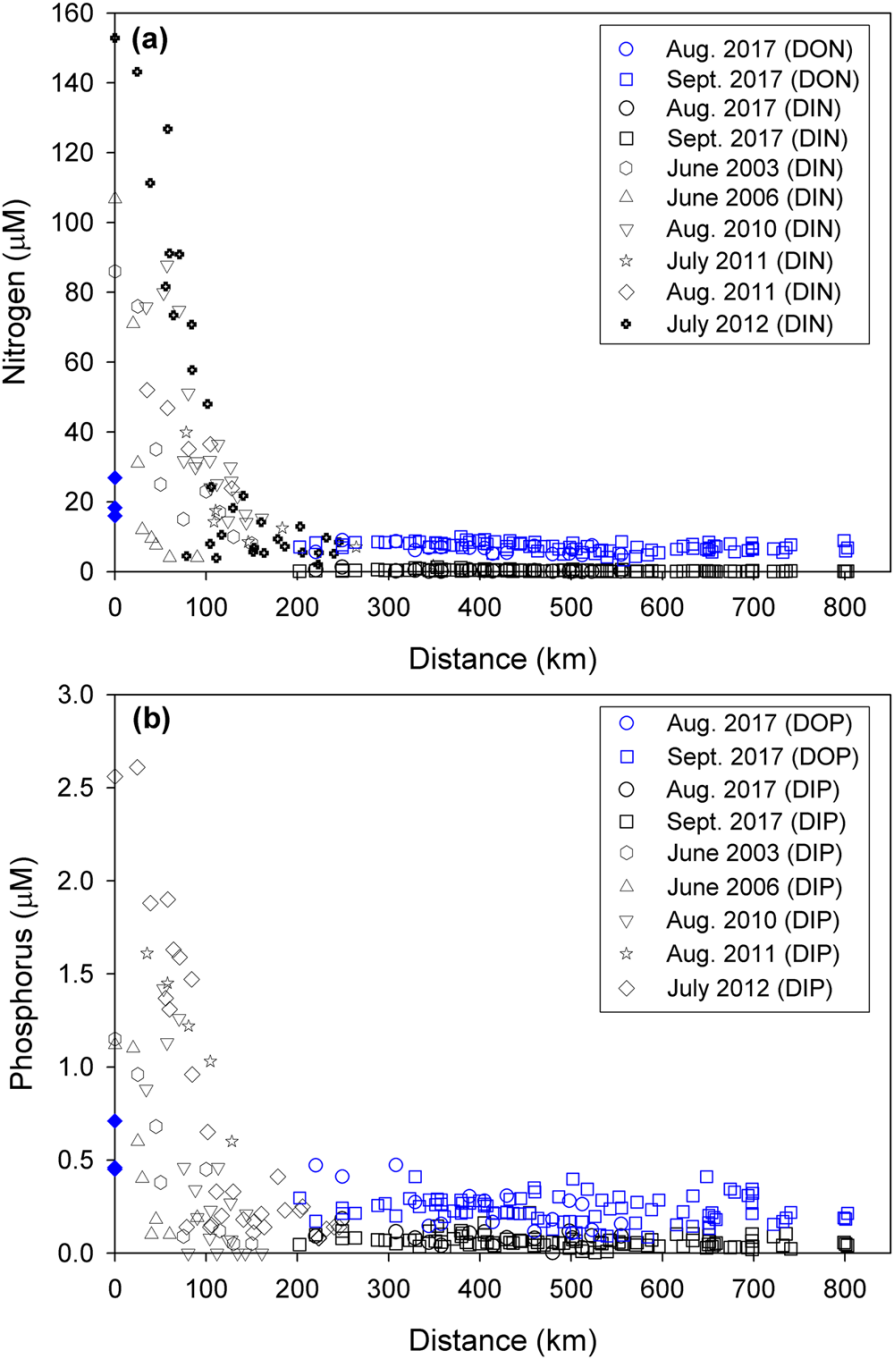
Distributions of (**a**) nitrogen (DIN and DON) and (**b**) phosphorus (DIP and DOP) in surface waters plotted against distance off the Changjiang estuary during the summer. The reference data between 2003 and 2012 are obtained from Chen *et al*.^17,18^ and Gao *et al*.^19^. The blue filled symbols denote DON and DOP concentrations in the Changjiang River obtained from Liu *et al*.^26,27^ and Shen and Liu^28^. The distances were calculated between Chongming Island (located in the Changjiang estuary) and each sampling station using the latitude and longitude of those points.

The concentrations of nutrients in the study region, including all previous data observed in this region, were plotted against the distance from the estuary (Fig. 3). It is clear that DIN and DIP concentrations decreased sharply from 153 µM and 2.6 µM to 0.16 µM and 0.05 µM, respectively, within 200 km from the estuary. They are consistently depleted from 200 to 800 km (Fig. 3). As such, DIN:DIP ratios decreased sharply from 65 to 3 and remained constant (5 ± 7) in the study region (Fig. 2). In contrast, the concentrations of DON (20 µM) and DOP (0.54 µM) were minor in the river water and decreased slightly within 200 km (perhaps due to vigorous mixing between river water and seawater). They were constantly high, relative to DIN and DIP, from 200 to 800 km. The ratios of DON:DOP were about 33 ± 11 (Fig. 2; Supplementary Table S1), similar to the values obtained previously from Changjiang River waters^26–28^, over the entire region (0–800 km). In the study region (200–800 km), the concentrations of DIN and DIP were lower than 1.3 µM (avg. 0.28 ± 0.26 µM) and 0.22 µM (avg. 0.07 ± 0.04 µM), respectively, which were much lower than the concentrations of DON (3.4–10.1 µM, avg. 7.0 ± 1.3 µM) and DOP (0.09–0.47 µM, avg. 0.24 ± 0.08 µM) (Figs. 2 and 3; Supplementary Table S1). This trend suggests that the contributions of DON and DOP to the TDN and TDP were dominant (about 96 ± 3% for TDN and about 78 ± 10% for TDP) in the study region, although they were minor in the source river water.

### Behaviors of dissolved nutrients during the long-range transport

**Figure 4:**
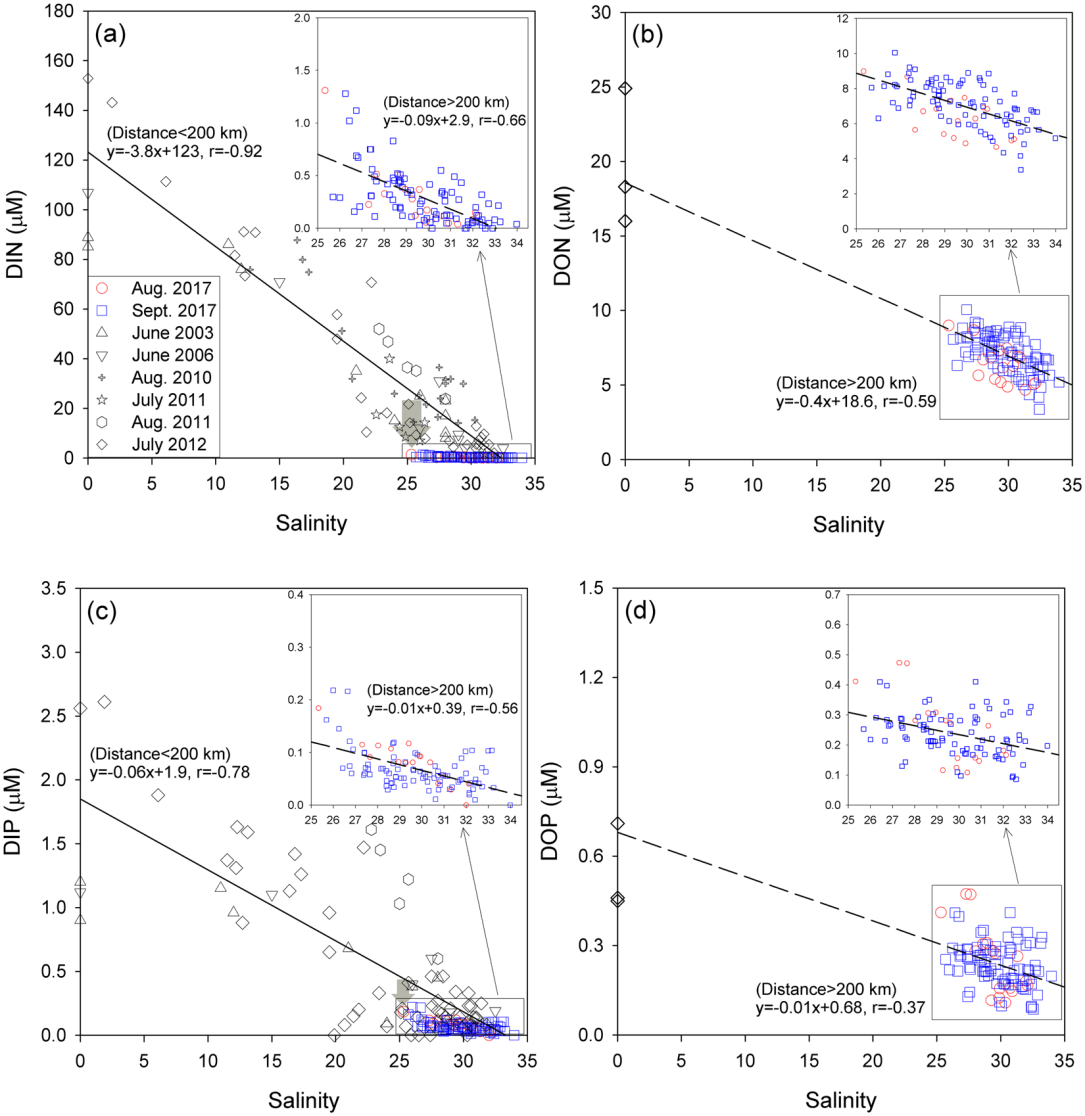
Scatterplots of (**a**) DIN, (**b**) DON, (**c**) DIP, and (**d**) DOP versus salinities in surface waters of the Changjiang estuary and East China Sea. The reference data between 2003 and 2012 are obtained from Chen *et al*.^17,18^, Gao *et al*.^19^, Liu *et al*.^26,27^, and Shen and Liu^28^. The solid lines indicate the relationship between salinity and inorganic nutrients from the Changjiang estuary (0 km) to 200 km. The dashed lines indicate the relationship between salinity and inorganic nutrients or organic nutrients from 200 to 800 km. The gray filled arrows denote the removed DIN and DIP from the expected concentrations by conservative mixing.

In order to understand the behaviors of nutrients over the long-range transport, the concentrations of DIN, DIP, DON, and DOP were plotted against salinities (Fig. 4). The concentrations of DIN and DIP show conservative mixing patterns for low-salinity waters (salinity < 20). They were then sharply removed and depleted in the study region, depending on the distance from the estuary, mainly between 100 km and 200 km (Figs. 3 and 4). The conservative mixing patterns in the low-salinity water have been reported by Yao *et al*.^16^ and Chen *et al*.^17^. Zhang^29^ attributed the conservative behavior of DIN and DIP in this zone to low primary production (chlorophyll *a* < 5 µg L^−1^) owing to high total suspended matter (up to 1300 mg L^−1^), causing limited light penetration, up to a distance of ~50 km off the river mouth. Zhang^29^ also showed a sharp increase in chlorophyll *a* concentration up to 20 µg L^−1^ at a distance of approximately 100 km off the Changjiang estuary, followed by a rapid decrease toward the open ocean. As such, the concentrations of DIN (0.28 ± 0.26 µM) and DIP (0.07 ± 0.04 µM) in the study region were much lower the expected concentrations of DIN (11.2 ± 6.7 µM) and DIP (0.16 ± 0.09 µM) from conservative mixing (Fig. 4). This seems to be associated with rapid consumption of DIN and DIP by phytoplankton. In general, the consumed nutrients are known to be mostly re-mineralized in the subsurface layer in this region^13^.

In contrast to DIN and DIP, the concentrations of DON (7.0 ± 1.3 µM) and DOP (0.24 ± 0.08 µM) measured in the study region were similar to the expected concentrations of DON (6.7 ± 0.8 µM) and DOP (0.38 ± 0.02 µM) from conservative mixing (Fig. 4). This result indicates that organic nutrients are conservatively mixed from the river mouth water to the offshore water up to 800 km (Figs. 3 and 4). The arrival time of CDW from the Changjiang River mouth to the vicinity of Jeju Island (~450 km) was estimated to be 20–35 days, based on the horizontal distribution of ^223^Ra activities^24^. During this transport time, the degradation of dissolved organic nutrients should be insignificant considering the fact that only approximately 7–11% of the riverine dissolved organic carbon (DOC) in seven river estuaries of the southeastern USA is decomposable in a 100 day timeframe due to the large portions of recalcitrant humic DOC^30,31^. Similarly, the production of dissolved organic nutrients might be insignificant (~10% relative to the DON change of ~14 µM) if the known production rate of DON (~2 nmol N L^−1^ h^−1^) in eutrophic oceans (i.e., Southern California Bight, shelf break of Atlantic off Spain, and Chilean upwelling region)^32–34^ is applied. In addition, the atmospheric deposition of DON by precipitation or vertical mixing seems to be negligible considering that the concentrations of DON in the surface water in the study region, precipitation^35^, and the subsurface water in this region^13^ are similar under a strong vertical stratification in summer. Thus, we conclude that the other sources and sinks cannot influence significantly the distributions of DON and DOP in the study region and perhaps are included in the scattering of the plots (Fig. 4).

In order to estimate the removal of nutrients in the mixing zone (0–800 km), we calculated the conservative mixing proportions between river water and pristine open-ocean seawater in the study region. The end-member concentrations of DIN, DIP, DON, and DOP were assumed to be 153 µM, 2.6 µM, 20 µM, and 0.54 µM for river waters^19,26–28^ and 0.04 µM, 0.004 µM, 5.2 µM, and 0.20 µM for open ocean waters from Fig. 4, respectively. In the study region, about 100% (99–100%) and 97% (92–100%) of riverine DIN and DIP were removed. However, the contribution of riverine DON and DOP to the total DON and DOP (including open ocean sources) were about 34% (17–49%) and 44% (16–88%), respectively, due to their conservative mixing. Therefore, riverine DON and DOP seem to be important for the transport of riverine nutrients to the open ocean and subsequent changes in nutrient conditions for ecosystem in the surface layer.

The proportions of DON and DOP against TDN and TDP observed in the study region were comparable to those observed in major oligotrophic oceans, such as the eastern subtropical North Pacific Ocean (DON: 5–6 µM, 96–99% of TDN, DOP: 0.1–0.35 µM, 75–90% of TDP)^36^, South Pacific Ocean (DON: 5.5–6 µM, 100% of TDN, DOP: 0.1–0.25 µM, 20–50% of TDP)^37^, and Atlantic Ocean (DON: 3.5–6.5 µM, 55–100% of TDN, DOP: 0.07–0.43 µM, 67–100% of TDP)^38^. Thus, the role of DON and DOP concentrations, together with their ratios, in the study region may be similar to that in other major oceans. For example, diatoms dominate in the inshore waters (salinity < 31 and DIN > 10 µM) due to the strong influence of the Changjiang plume in the East China Sea, while the density of dinoflagellates increases gradually with distance away from the diatom dominated zone^20^. This could happen because many dinoflagellate species are capable of taking up organic nutrients^39,40^, although this process is limited for diatoms^39,41^.

## Conclusions

The concentrations of riverine DIN and DIP decreased sharply from the Changjiang River estuary and depleted within 200 km from the river mouth, and the concentrations of DIN and DIP were about 0.28 µM and 0.07 µM, respectively, between 200 and 800 km. This seems to be associated with rapid consumption of inorganic nutrients by phytoplankton. In contrast, DON and DOP exhibited conservative mixing behaviors up to 800 km, and their concentrations were about 7.0 µM and 0.24 µM, respectively, between 200 and 800 km. Therefore, DON and DOP account for about 96% and 78% of TDN and TDP, respectively, between 200 and 800 km, although their proportions were minor in the river water. Our results suggest that dissolved organic nutrients play a critical role on riverine nutrient transport to the surface waters of the open ocean. Further studies are necessary to determine the role of riverine nutrient composition changes on ecosystem changes in the remote oceans.

## Electronic supplementary material

Supplementary Information

## Data Availability

The datasets analysed during the current study are available from the corresponding author on reasonable request.
