# Supplementary material for: Significant and conservative long-range transport of dissolved organic nutrients in the Changjiang diluted water
Source: Sci Rep. 2018 Aug 24;8:12768. doi: 10.1038/s41598-018-31105-1 (PMC6109133; doi:10.1038/s41598-018-31105-1)
Supplement: Supplementary file 1 — Supplementary Information [file 41598_2018_31105_MOESM1_ESM.docx]

**Supplementary Information**

**Significant and conservative long-range transport of dissolved organic nutrient in the Changjiang diluted water**

Hyeong Kyu Kwon^1^, Guebuem Kim^1^*, Jeomshik Hwang^1^, Weol Ae Lim^2^, Jong Woo Park^2^ & Tae Hoon Kim^3^

^1^School of Earth and Environmental Sciences/Research Institute of Oceanography, Seoul National University, Seoul 08826, Republic of Korea

^2^Ocean Climate and Ecology Research Division, National Institute of Fisheries Science, Busan 46083, Republic of Korea

^3^Department of Earth and Marine Sciences, Jeju National University, Jeju 63243, Republic of Korea

^*^Corresponding author: Guebuem Kim (gkim@snu.ac.kr)

**Table S1.** The concentrations of salinity and dissolved nutrients in surface waters of the East China Sea during the summer 2017. (ND=non-detect)

| **Period** | **Latitude** | **Longitude** | **Salinity** | **Dissolved nutrients (µM)** | | | | **DIN:**  **DIP** | **DON:**  **DOP** |
| --- | --- | --- | --- | --- | --- | --- | --- | --- | --- |
|  |  |  |  | **DIN** | **DIP** | **DON** | **DOP** |  |  |
| Aug.  16-19,  2017 | 33º24'04'' | 124º24'00'' | 30.91 | 0.05 | 0.057 | 6.8 | 0.15 | 0.8 | 47 |
|  | 33º24'04'' | 124º24'00'' | 32.12 | 0.15 | 0.040 | 5.1 | 0.17 | 3.7 | 31 |
|  | 33º24'04'' | 126º00'00'' | 29.27 | 0.28 | 0.080 | 7.3 | 0.12 | 3.5 | 63 |
|  | 33º38'03'' | 126º21'03'' | 30.37 | 0.09 | 0.081 | 6.3 | 0.11 | 1.1 | 58 |
|  | 33º35'08'' | 127º03'03'' | 29.93 | 0.17 | 0.092 | 4.9 | 0.16 | 1.9 | 31 |
|  | 33º00'00'' | 125º00'00'' | 30.73 | 0.12 | 0.041 | 6.9 | 0.16 | 2.9 | 44 |
|  | 33º00'00'' | 125º54'00'' | 28.95 | 0.37 | 0.082 | 5.4 | 0.31 | 4.5 | 18 |
|  | 33º00'00'' | 126º30'00'' | 32.00 | 0.08 | ND | 5.0 | 0.18 | 41 | 28 |
|  | 33º00'00'' | 127º00'00'' | 29.88 | 0.06 | 0.093 | 7.5 | 0.12 | 0.6 | 60 |
|  | 32º30'00'' | 124º00'00'' | 25.33 | 1.31 | 0.184 | 9.0 | 0.41 | 7.1 | 22 |
|  | 32º30'00'' | 125º00'00'' | 29.58 | 0.37 | 0.081 | 6.2 | 0.27 | 4.5 | 22 |
|  | 32º30'00'' | 125º53'01'' | 28.02 | 0.33 | 0.113 | 6.7 | 0.28 | 2.9 | 24 |
|  | 32º30'00'' | 127º05'01'' | 31.32 | 0.04 | 0.029 | 4.7 | 0.26 | 1.3 | 18 |
|  | 32º00'00'' | 124º00'00'' | 27.65 | 0.51 | 0.091 | 5.6 | 0.47 | 5.6 | 12 |
|  | 32º00'00'' | 125º00'00'' | 27.30 | 0.23 | 0.115 | 8.7 | 0.47 | 2.0 | 18 |
|  | 32º00'00'' | 125º53'01'' | 28.61 | 0.51 | 0.107 | 6.8 | 0.31 | 4.7 | 22 |
|  | 32º00'00'' | 127º04'01'' | 29.40 | 0.12 | 0.117 | 5.2 | 0.28 | 1.0 | 18 |
| Aug. 22-  Sept. 6,  2017 | 32º30'00'' | 127º23'06'' | 32.41 | 0.32 | 0.010 | 4.2 | 0.09 | 35.6 | 45 |
|  | 32º30'00'' | 127º05'06'' | 30.62 | 0.35 | 0.011 | 5.5 | 0.17 | 31.8 | 33 |
|  | 32º30'00'' | 126º47'60'' | 28.82 | 0.39 | 0.053 | 6.4 | 0.30 | 7.4 | 21 |
|  | 32º30'00'' | 126º30'00'' | 28.65 | 0.43 | 0.042 | 7.4 | 0.35 | 10.2 | 21 |
|  | 32º30'00'' | 126º12'00'' | 27.37 | 0.75 | 0.057 | 8.9 | 0.29 | 13.2 | 31 |
|  | 32º30'00'' | 125º53'06'' | 27.40 | 0.75 | 0.099 | 9.2 | 0.26 | 7.6 | 35 |
|  | 32º30'00'' | 125º35'13'' | 26.74 | 1.12 | 0.121 | 10.1 | 0.28 | 9.3 | 35 |
|  | 32º30'00'' | 125º17'12'' | 26.25 | 1.28 | 0.145 | 8.1 | 0.29 | 8.8 | 28 |
|  | 32º30'00'' | 125º00'00'' | 26.43 | 1.02 | 0.070 | 8.8 | 0.41 | 14.6 | 22 |
|  | 32º30'00'' | 124º30'00'' | 27.45 | 0.56 | 0.071 | 8.5 | 0.26 | 7.9 | 33 |
|  | 32º30'00'' | 126º00'00'' | 28.73 | 0.44 | 0.119 | 6.9 | 0.24 | 3.7 | 28 |
|  | 32º00'00'' | 127º22'12'' | 33.99 | 0.04 | ND | 5.2 | 0.20 | 10.0 | 26 |
|  | 32º00'00'' | 127º04'06'' | 30.32 | 0.09 | 0.027 | 7.0 | 0.17 | 3.3 | 41 |
|  | 32º00'00'' | 126º46'06'' | 30.26 | 0.16 | 0.044 | 7.2 | 0.17 | 3.6 | 42 |
|  | 32º00'00'' | 126º28'60'' | 28.91 | 0.47 | 0.068 | 7.9 | 0.21 | 6.9 | 37 |
|  | 32º00'00'' | 126º10'60'' | 28.61 | 0.52 | 0.041 | 8.6 | 0.21 | 12.7 | 40 |
|  | 32º00'00'' | 125º53'06'' | 28.40 | 0.48 | 0.046 | 8.4 | 0.27 | 10.4 | 31 |
|  | 32º00'00'' | 125º35'06'' | 28.73 | 0.51 | 0.034 | 7.9 | 0.20 | 15.0 | 40 |
|  | 32º00'00'' | 125º17'06'' | 28.40 | 0.83 | 0.036 | 8.5 | 0.25 | 23.1 | 34 |
|  | 32º00'00'' | 125º00'00'' | 29.06 | 0.34 | 0.051 | 8.6 | 0.20 | 6.7 | 43 |
|  | 32º00'00'' | 124º30'00'' | 29.25 | 0.32 | 0.082 | 8.4 | 0.21 | 3.9 | 39 |
|  | 32º00'00'' | 124º00'00'' | 29.69 | 0.26 | 0.099 | 8.3 | 0.17 | 2.6 | 48 |
|  | 31º30'00'' | 127º04'06'' | 31.16 | 0.49 | 0.055 | 5.0 | 0.17 | 8.9 | 30 |
|  | 31º30'00'' | 126º46'06'' | 30.35 | 0.13 | 0.058 | 5.9 | 0.19 | 2.2 | 31 |
|  | 31º30'00'' | 126º28'60'' | 28.82 | 0.12 | 0.054 | 8.2 | 0.21 | 2.2 | 39 |
|  | 31º30'00'' | 126º10'60'' | 28.52 | 0.20 | 0.060 | 7.9 | 0.25 | 3.3 | 31 |
|  | 31º30'00'' | 125º53'06'' | 28.73 | 0.19 | 0.091 | 7.6 | 0.21 | 2.1 | 35 |
|  | 31º30'00'' | 125º35'06'' | 28.40 | 0.27 | 0.065 | 8.3 | 0.26 | 4.2 | 32 |
|  | 31º30'00'' | 125º17'06'' | 28.15 | 0.42 | 0.069 | 8.1 | 0.29 | 6.1 | 28 |
|  | 31º30'00'' | 125º00'00'' | 28.24 | 0.32 | 0.069 | 8.3 | 0.27 | 4.6 | 31 |
|  | 31º30'00'' | 124º30'00'' | 28.71 | 0.20 | 0.079 | 7.6 | 0.21 | 2.5 | 36 |
|  | 31º30'00'' | 124º00'00'' | 29.62 | 0.16 | 0.046 | 7.1 | 0.30 | 3.5 | 24 |

**Table S1.** Continued.

| **Period** | **Latitude** | **Longitude** | **Salinity** | **Dissolved nutrients (µM)** | | | | **DIN:**  **DIP** | **DON:**  **DOP** |
| --- | --- | --- | --- | --- | --- | --- | --- | --- | --- |
|  |  |  |  | **DIN** | **DIP** | **DON** | **DOP** |  |  |
| Aug. 22-  Sept. 6,  2017 | 34º01'60'' | 126º34'00'' | 29.60 | 0.03 | 0.068 | 6.0 | 0.24 | 0.4 | 25 |
|  | 33º56'36'' | 126º31'24'' | 29.19 | 0.36 | 0.051 | 6.1 | 0.28 | 7.0 | 22 |
|  | 33º47'30'' | 126º26'18'' | 28.46 | 0.45 | 0.029 | 7.3 | 0.34 | 15.7 | 21 |
|  | 33º38'18'' | 126º21'18'' | 26.74 | 0.69 | 0.074 | 8.2 | 0.40 | 9.3 | 21 |
|  | 34º17'60'' | 127º31'59'' | 27.58 | 0.11 | 0.068 | 7.8 | 0.14 | 1.6 | 55 |
|  | 34º20'48'' | 127º43'54'' | 32.89 | ND | 0.063 | 6.8 | 0.13 | 0.1 | 51 |
|  | 34º13'42'' | 127º35'24'' | 33.16 | 0.22 | 0.104 | 6.7 | 0.31 | 2.1 | 22 |
|  | 34º06'42'' | 127º26'48'' | 31.97 | 0.05 | 0.102 | 6.4 | 0.15 | 0.5 | 42 |
|  | 33º54'06'' | 127º15'12'' | 32.71 | 0.23 | 0.071 | 6.2 | 0.23 | 3.3 | 27 |
|  | 33º45'30'' | 127º09'12'' | 30.71 | 0.12 | 0.056 | 7.0 | 0.27 | 2.1 | 26 |
|  | 33º35'48'' | 127º03'12'' | 30.56 | 0.22 | 0.051 | 8.6 | 0.24 | 10.8 | 36 |
|  | 34º25'00'' | 127º40'00'' | 31.71 | ND | 0.047 | 6.2 | 0.16 | 0.2 | 38 |
|  | 34º22'18'' | 127º48'30'' | 30.17 | ND | 0.054 | 7.2 | 0.18 | 0.4 | 40 |
|  | 34º15'00'' | 127º52'06'' | 30.82 | 0.13 | 0.045 | 7.4 | 0.19 | 2.8 | 39 |
|  | 34º05'30'' | 127º56'54'' | 32.72 | 0.05 | 0.035 | 5.6 | 0.22 | 1.4 | 26 |
|  | 33º51'30'' | 128º03'00'' | 29.80 | 0.08 | 0.096 | 8.5 | 0.20 | 0.8 | 42 |
|  | 33º37'18'' | 128º09'12'' | 30.75 | 0.26 | 0.029 | 6.4 | 0.41 | 8.9 | 16 |
|  | 34º37'60'' | 128º25'00'' | 31.79 | 0.06 | 0.089 | 6.5 | 0.15 | 0.7 | 42 |
|  | 34º35'30'' | 128º34'30'' | 32.42 | 0.08 | 0.042 | 5.5 | 0.17 | 2.0 | 33 |
|  | 34º29'36'' | 128º41'12'' | 32.27 | 0.12 | 0.104 | 6.8 | 0.19 | 1.2 | 36 |
|  | 34º22'24'' | 128º49'42'' | 32.13 | 0.20 | 0.023 | 7.7 | 0.22 | 8.6 | 35 |
|  | 35º01'18'' | 129º07'12'' | 30.98 | 0.12 | 0.057 | 8.9 | 0.19 | 2.1 | 47 |
|  | 34º57'36'' | 129º11'12'' | 31.78 | 0.03 | 0.049 | 6.0 | 0.18 | 0.7 | 33 |
|  | 34º53'54'' | 129º15'24'' | 32.18 | 0.12 | 0.042 | 6.8 | 0.21 | 3.0 | 32 |
|  | 33º24'24'' | 126º00'00'' | 30.60 | 0.34 | 0.074 | 8.5 | 0.33 | 4.5 | 26 |
|  | 33º24'24'' | 125º47'60'' | 30.73 | 0.32 | 0.064 | 8.2 | 0.29 | 5.0 | 28 |
|  | 33º24'24'' | 125º35'60'' | 31.48 | 0.43 | 0.074 | 7.3 | 0.22 | 5.9 | 33 |
|  | 33º24'24'' | 125º24'00'' | 31.05 | 0.34 | 0.055 | 5.2 | 0.22 | 6.2 | 24 |
|  | 33º24'24'' | 125º12'00'' | 30.04 | 0.42 | 0.051 | 8.9 | 0.28 | 8.2 | 32 |
|  | 33º24'24'' | 125º00'00'' | 27.65 | 0.45 | 0.058 | 9.1 | 0.22 | 7.7 | 41 |
|  | 33º24'24'' | 124º47'60'' | 27.57 | 0.49 | 0.077 | 7.5 | 0.23 | 6.4 | 33 |
|  | 33º24'24'' | 124º35'60'' | 26.82 | 0.67 | 0.060 | 8.1 | 0.26 | 11.2 | 31 |
|  | 33º24'24'' | 124º24'00'' | 28.69 | 0.45 | 0.117 | 7.1 | 0.29 | 3.8 | 25 |
|  | 33º00'00'' | 127º42'00'' | 32.57 | ND | 0.041 | 4.8 | 0.09 | 0.1 | 56 |
|  | 33º00'00'' | 127º24'00'' | 32.43 | 0.03 | 0.049 | 3.4 | 0.10 | 0.6 | 35 |
|  | 33º00'00'' | 127º05'60'' | 30.08 | 0.06 | 0.059 | 6.0 | 0.10 | 1.0 | 61 |
|  | 33º00'00'' | 126º47'60'' | 29.92 | 0.10 | 0.064 | 6.5 | 0.11 | 1.5 | 59 |
|  | 33º00'00'' | 126º30'00'' | 27.43 | 0.31 | 0.060 | 7.3 | 0.13 | 5.1 | 57 |
|  | 33º00'00'' | 126º12'00'' | 26.65 | 0.16 | 0.216 | 7.2 | 0.21 | 0.7 | 34 |
|  | 33º00'00'' | 125º54'00'' | 26.00 | 0.29 | 0.218 | 6.3 | 0.22 | 1.3 | 29 |
|  | 33º00'00'' | 125º35'60'' | 25.69 | 0.30 | 0.162 | 8.1 | 0.25 | 1.8 | 32 |
|  | 33º00'00'' | 125º17'60'' | 26.89 | 0.21 | 0.106 | 6.9 | 0.27 | 1.9 | 26 |
|  | 33º00'00'' | 125º00'00'' | 27.41 | 0.31 | 0.100 | 7.0 | 0.29 | 3.1 | 25 |
|  | 34º36'00'' | 128º04'60'' | 32.31 | 0.06 | 0.030 | 6.0 | 0.31 | 2.0 | 20 |
|  | 34º04'36'' | 128º30'00'' | 31.36 | 0.27 | 0.034 | 6.4 | 0.32 | 8.0 | 20 |
|  | 34º13'30'' | 128º24'00'' | 29.62 | 0.23 | 0.066 | 8.2 | 0.27 | 3.5 | 30 |
|  | 34º22'12'' | 128º18'24'' | 32.99 | 0.08 | 0.103 | 6.7 | 0.21 | 0.8 | 31 |
|  | 34º31'00'' | 128º12'12'' | 32.13 | 0.07 | 0.020 | 6.9 | 0.34 | 3.4 | 20 |
|  | 34º27'00'' | 128º00'30'' | 33.23 | 0.06 | 0.033 | 5.6 | 0.33 | 1.9 | 17 |
|  | 33º44'18'' | 128º23'54'' | 31.25 | 0.09 | 0.030 | 8.0 | 0.34 | 3.0 | 23 |

**Table S1.** Continued.

| **Period** | **Latitude** | **Longitude** | **Salinity** | **Dissolved nutrients (µM)** | | | | **DIN:**  **DIP** | **DON:**  **DOP** |
| --- | --- | --- | --- | --- | --- | --- | --- | --- | --- |
|  |  |  |  | **DIN** | **DIP** | **DON** | **DOP** |  |  |
| Aug. 22-  Sept. 6,  2017 | 33º35'36'' | 127º51'54'' | 29.23 | 0.11 | 0.037 | 7.3 | 0.22 | 2.9 | 33 |
|  | 33º33'36'' | 127º34'00'' | 30.82 | 0.05 | 0.038 | 5.4 | 0.29 | 1.3 | 19 |
|  | 33º31'42'' | 127º16'48'' | 31.64 | ND | 0.044 | 4.3 | 0.16 | 0.2 | 27 |
|  | 33º30'18'' | 127º04'30'' | 31.99 | 0.05 | 0.060 | 5.3 | 0.30 | 0.9 | 18 |
